# Advanced endometrial cancer: New medical treatment options on the horizon

**DOI:** 10.1111/aogs.14499

**Published:** 2023-01-31

**Authors:** Ingvild Vistad, Line Bjørge

**Affiliations:** ^1^ Department of Gynecology and Obstetrics Sørlandet Hospital Kristiansand Norway; ^2^ Clinical Department University of Bergen Bergen Norway; ^3^ Centre for Cancer Biomarkers CCBIO, Department of Clinical Science University of Bergen Bergen Norway; ^4^ Department of Obstetrics Gynecology Haukeland University Hospital Bergen Norway

Endometrial cancer (EC) is the most common gynecologic malignancy in the developed world and the sixth most common cancer in women worldwide.^1^ The steady increase in EC incidence rates over the last decades is primarily attributed to the worldwide epidemic of obesity.^2^ Surgery is the primary treatment. While most patients with early‐stage and lowrisk disease require no additional treatment, radiotherapy and chemotherapy, alone or combined, are often indicated in women with a high risk of recurrence.^3^ The role of adjuvant radiotherapy is debatable in intermediate‐risk cases. While European guidelines recommend adjuvant brachytherapy to decrease vaginal recurrence,^3^ the Nordic strategy is to minimize adjuvant radiotherapy because its efficacy on overall survival has not been proven.^4^

Carboplatin plus paclitaxel is the current standard chemotherapy for first‐line management of advanced, recurrent and metastatic EC.^3^ Unfortunately, many patients relapse after treatment with this combination. Hormonal treatment is an alternative option in a subset of patients with hormone‐receptor positive‐tumors, although most patients will require additional systemic treatment.^5^ Numerous studies have explored whether chemotherapy results in prolonged survival, but many have included both high‐ and low‐risk patients often combined with radiotherapy in one or both arms, which makes it difficult to interpret the results. In addition, in most studies the results are negative.^6^

Traditionally, the selection of adjuvant treatment in EC is based on stage, histological subtype, histological grade of differentiation, and a few histopathological prognostic factors such as lymphovascular space invasion. However, histopathological diagnostics have shortcomings, and the interrater reliability among pathologists is as low as 30%.^7^ To overcome these challenges, numerous molecular and histological biomarkers have been investigated to determine their ability to function as predictors of recurrence and prognosis. In 2013, the Cancer Genome Atlas (TCGA) project published analyses of the genomes of 373 EC cases.^8^ Four molecular subtypes of EC were identified: polymerase epsilon (POLE) ultramutated, microsatellite instability hypermutated (MSI‐H)/mismatch repair–deficient subtype (MMRd), copy number low, and copy number high.^8^ In routine clinical practice, molecular classification is based on immunohistochemistry for MMR expression and p53, and next‐generation sequencing for POLE mutated (POLEmut) tumors.^3^ Cases where information for classification is insufficient are referred to as having no specific molecular profile and are included in the copy number low subtype. Stratification of clinical cohorts into the four molecular subtypes has revealed distinct differences in prognoses.^3^, ^9^

The POLEmut subtype constitutes 5%–10% of EC cases.^6^ These tumors are characterized by a very high mutation frequency, high tumor infiltrating lymphocytes, and low copy number alterations. POLEmut tumors have an excellent prognosis and may not require adjuvant treatment.^3^ The MMRd subtype constitutes 25% of EC cases. Their profile is similar to POLEmut tumors, with a high mutational burden, extensive infiltration of lymphocytes, and low copy number alterations, but mutations are present in different genes and the prognosis is intermediate. Copy number low is the most predominant subtype observed and constitutes around 40% of EC cases with a low mutational burden and frequent expression of hormone‐receptors.^6^ This group is heterogeneous, and the prognosis is variable. Copy number high is the fourth subtype, observed in 25%–30% of EC cases.^6^ This subtype, which includes serous EC, has the worst prognosis and is associated with mutations in the *TP53* gene in >90% of cases. The different EC subtypes are also associated with pathogenic variants of well‐known genes, such as *PTEN*, *PIK3CA*, *KRAS*, and *ARID1A*, amplification of *Erb2*, and overexpression of PD‐L1.

Clinical studies analyzing the effect of immune checkpoint inhibitors alone or in combination with various targeted agents, such as lenvatinib and poly (ADP‐ribose) polymerase inhibitors (PARPi), have been shown to be effective in selected subgroups. Anti‐human epidermal growth factor receptor 2 (HER2) is another promising agent in the serous EC subgroup. Several ongoing phase III studies evaluating the efficacy of immune checkpoint inhibitors and chemotherapy and/or PARPi (eg ENGOT‐EN6/NSGO‐RUBY and GOG‐3041/DUO‐E) and lenvatinib plus pembrolizumab (LEAP‐001) are presumed to be practice‐changing and will be included in the international recommendations for standard of care.

The use of molecular testing for EC at diagnosis in Nordic countries is still in its infancy, and the gynecological oncological environment needs training to be able to use the new information. Furthermore, as the primary treatment of EC will be more challenging in the future because of its heterogeneity, treatment decisions should be made in multidisciplinary teams. Recurrent EC is often resistant to chemotherapy, so there is a need to develop more efficient therapeutics. It is our hope that molecular classification, in combination with selected biomarkers, can also be used as a tool for treatment selection in the recurrent setting. While the American Food and Drug Administration and the European Medicines Agency have approved the use of immune checkpoint inhibitors in patients with recurrent or advanced MMRd EC, the health authorities in most Nordic countries are still awaiting the results of ongoing phase III studies.

Sustained estrogen exposure is a significant risk factor for EC, and the number of women diagnosed with EC is expected to increase. Therefore, focusing on prevention in terms of education and lifestyle interventions is still essential to reduce the incidence of the disease.^2^ Furthermore, until we have better information about the pathogenesis and strategies for early detection, EC will continue to be diagnosed in advanced stages, or some patients will fail their primary treatment and experience recurrence. Implementation of molecular‐based classification for risk stratification and treatment selection is only just beginning. We hope that implementation of the new molecular‐guided treatment algorithm will soon result in improved management of EC.
